# Voluntary Exercise Prevents Cisplatin-Induced Muscle Wasting during Chemotherapy in Mice

**DOI:** 10.1371/journal.pone.0109030

**Published:** 2014-09-30

**Authors:** Pernille Hojman, Jonas Fjelbye, Bo Zerahn, Jesper F. Christensen, Christine Dethlefsen, Camilla K. Lonkvist, Claus Brandt, Hanne Gissel, Bente Klarlund Pedersen, Julie Gehl

**Affiliations:** 1 The Centre of Inflammation and Metabolism and the Centre for Physical Activity Research, Department of Infectious Diseases, Rigshospitalet, University of Copenhagen, Copenhagen, Denmark; 2 Department of Clinical Physiology and Nuclear Medicine, Copenhagen University Hospital Herlev, Herlev, Denmark; 3 Copenhagen University Hospital, The University Hospitals Centre for Health Care Research (UCSF), Copenhagen, Denmark; 4 Department of Oncology, Copenhagen University Hospital Herlev, Herlev, Denmark; 5 Institute of Biomedicine, University of Aarhus, Aarhus C, Denmark; University of Rome La Sapienza, Italy

## Abstract

Loss of muscle mass related to anti-cancer therapy is a major concern in cancer patients, being associated with important clinical endpoints including survival, treatment toxicity and patient-related outcomes. We investigated effects of voluntary exercise during cisplatin treatment on body weight, food intake as well as muscle mass, strength and signalling. Mice were treated weekly with 4 mg/kg cisplatin or saline for 6 weeks, and randomized to voluntary wheel running or not. Cisplatin treatment induced loss of body weight (29.8%, P<0.001), lean body mass (20.6%, P = 0.001), as well as anorexia, impaired muscle strength (22.5% decrease, P<0.001) and decreased glucose tolerance. In addition, cisplatin impaired Akt-signalling, induced genes related to protein degradation and inflammation, and reduced muscle glycogen content. Voluntary wheel running during treatment attenuated body weight loss by 50% (P<0.001), maintained lean body mass (P<0.001) and muscle strength (P<0.001), reversed anorexia and impairments in Akt and protein degradation signalling. Cisplatin-induced muscular inflammation was not prevented by voluntary wheel running, nor was glucose tolerance improved. Exercise training may preserve muscle mass in cancer patients receiving cisplatin treatment, potentially improving physical capacity, quality of life and overall survival.

## Background

Loss of muscle mass is a common clinical finding across cancer diagnoses and stages attributable to a range of factors related to both anti-cancer treatment, patient lifestyle and the cancer disease itself [1]. In both patients with early and advanced stage disease, muscle mass significantly impacts patient-reported and clinical outcomes, including survival and disease progression.

Cisplatin is a cornerstone in curative and adjuvant treatment of several solid tumours including testicular-, head and neck-, uterine cervix and lung cancer [2]–[4]. Cisplatin is highly effective but also associated with plethora of adverse reactions including nausea, anorexia, dysphagia, pain, and fatigue, all of which may be associated with muscular dysfunction. Studies in muscle cell culture suggest that cisplatin can induce atrophy-related genes, proteosomal proteolysis and inflammation in muscle cells [5], [6].

Currently, there is emerging enthusiasm for exercise interventions in cancer patients due to accumulating evidence of beneficial effects on fitness, body composition, muscle strength, functional performance and patient reported quality of life [7]–[9]. Structured exercise training induces a wide range of biochemical alterations, which, under normal circumstances, improve the contractile, metabolic and endocrine properties of skeletal muscle [10]. However, these exercise-induced adaptations may be affected by concomitant influence of cisplatin. For the anthracycline, doxorubicin, exercise has been shown to reverse doxorubicin-induced oxidative stress by induction of muscular antioxidant enzymes and heat shock protein 72 [11]. Evidence of such direct protective mechanisms of exercise remains to be determined for other chemotherapeutics including cisplatin.

Thus, we propose that voluntary wheel running during cisplatin treatment may ameliorate cisplatin-induced adverse effects on muscle mass and function in mice. Specifically, we investigated the effect of voluntary exercise during cisplatin treatment on body weight, food intake as well as muscle mass, strength and signalling. Moreover, we tested if there was an influence on results of anti-emetic treatment, and if exercise during recovery from cisplatin treatment could augment muscle mass restoration.

## Materials and Methods

### Animals and ethical considerations

All animal experiments were conducted in accordance with the recommendations of the European Convention for the Protection of Vertebrate Animals used for Experimentation and after approval of the experimental protocol by the Danish Animal Experiments Inspectorate. All animal experiments were performed according to the ARRIVE guidelines (Checklist S1). To ensure animal welfare, cisplatin treatment was discontinued if body weight fell below 20 g, for completion rates, please see Table 1. Eight-to-twelve week old female NMRI mice (own breed, FELASA tested) were housed in a temperature- and humidity-controlled room and maintained on a 12:12-h light-dark cycle with food and water *ad libitum*, with 3 mice per cage.

**Table 1:** Treatment completion rates.

Group	Treatment	Week 1	Week 2	Week 3	Week 4	Week 5	Week 6
**Study 1**							
**1**	CON	100% (16/16)	100% (16/16)	100% (16/16)	100% (16/16)	100% (16/16)	100% (16/16)
**2**	CIS	100% (16/16)	100% (16/16)	100% (16/16)	100% (16/16)	87% (14/16)	87% (14/16)
**3**	CON + EX	100% (18/18)	100% (18/18)	100% (18/18)	100% (18/18)	100% (18/18)	100% (18/18)
**4**	CIS + EX	100% (18/18)	100% (18/18)	100% (18/18)	100% (18/18)	100% (18/18)	100% (18/18)
**Study 2**							
**1**	CON	100% (9/9)	100% (9/9)	100% (9/9)	100% (9/9)	100% (9/9)	100% (9/9)
**2**	CIS	100% (10/10)	100% (10/10)	100% (10/10)	90% (9/10)	80% (8/10)	80% (8/10)
**3**	CIS + ONS	100% (10/10)	100% (10/10)	100% (10/10)	90% (9/10)	90% (9/10)	80% (9/10)
**4**	CIS + STER	100% (9/9)	100% (9/9)	100% (9/9)	88% (8/9)	88% (8/9)	78% (7/9)
**5**	CIS + STER + EX	100% (10/10)	100% (10/10)	100% (10/10)	100% (10/10)	100% (10/10)	90% (9/10)
**6**	CIS + EX	100% (10/10)	100% (10/10)	100% (10/10)	100% (10/10)	100% (10/10)	100% (10/10)
**Study 3**							
**1**	CON	100% (9/9)	100% (9/9)	100% (9/9)	100% (9/9)	100% (9/9)	100% (9/9)
**2**	CIS	100% (9/9)	100% (9/9)	100% (9/9)	100% (9/9)	100% (9/9)	100% (9/9)
**3**	CIS + EX in recovery	100% (9/9)	100% (9/9)	100% (9/9)	100% (9/9)	100% (9/9)	100% (9/9)

Percentage and number of mice completing cisplatin treatment. CON: Saline treated mice, CIS: cisplatin treated mice, ONS: Onsedansetron, STER: steroids, dexamethasone, EX: access to running wheels, and CIS + EX in recovery: access to running wheels in the 6 weeks recovery phase.

In study 1 in each group, the mice were divided between termination at rest or after 1 hour of acute exercise (swimming). Both groups had the following measurements made: body weight, food intake, weight of organs at termination and blood sampling at termination. In addition, the rest group were DXA scanned, performed the hang tests and had a glucose tolerance test made.

### Cisplatin treatment and voluntary exercise

Mice were randomised to receive weekly cisplatin 4 mg/kg (Hospira Nordic, Sweden) or to saline-control for a duration of 6 weeks. Following the initial group allocation, the mice were further randomized to cages with running wheels or not. Average running distances for experiment 3 are reported in Fig S1.

#### Experiment 1: Effect of cisplatin and exercise

Sixty-eight mice were randomly assigned to one of four groups: control sedentary (CON, n = 16), control exercise (CON+EX, n = 16), cisplatin sedentary (CIS, n = 18) and cisplatin exercise (CIS+EX, n = 18). All mice were weekly evaluated for body weight and food intake, and upon completion of the six-week period were measured as follows: body weight, weight of organs and blood sampling at termination. In addition, half of the mice were DXA scanned, performed hang tests and had a glucose tolerance test made, before they were euthanized at rest. The other half of each study group performed an acute swimming bout in 35°C water prior to euthanization.

#### Experiment 2: effect of antiemetic drugs

Fifty-eight mice were randomized to one of six groups, three of which were identical to groups in experiment one: control sedentary (CON, n = 9), cisplatin sedentary (CIS, n = 10), cisplatin exercise (CIS+EX, n = 10) and three groups given antiemetic drugs by i.p. injection for 3 days concomitant with each of the 6 weekly cisplatin treatments: cisplatin sedentary+ondansetron 1 mg/kg (Fresenius Kabi) (CIS+ondansetron, n = 10), cisplatin+sedentary+dexamethasone 5 mg/kg (Galen) (CIS+DEXA, n = 9) or cisplatin +dexametasone+exercise (CIS +DEXA+EX, n = 10). Three days before the first and 7 days after the last cisplatin treatment, all mice were DXA scanned. In addition, body weight and food intake were monitored weekly, and blood samples were collected at termination, 7 days after last cisplatin administration.

#### Experiment 3: effect of exercise on muscular recovery from cisplatin treatment

To evaluate recovery, we followed mice for a 6-wk pretreatment period (pre-CIS), a 6-wk treatment period (CIS) and a 6-wk follow-up period (recovery). Twenty-seven mice were randomized to one of three groups: saline-control sedentary (CON, n = 9), cisplatin sedentary (CIS, n = 9) or cisplatin exercise in the recovery period (CIS+Ex, n = 9). All mice were evaluated weekly for body mass and food intake, and further evaluated for body composition (DXA scan) prior to the treatment period, immediately after cisplatin-treatment and after the follow-up period.

### DXA scanning

Assessment of lean body mass (LBM) and fat mass (FM) were performed using a LUNAR Prodigy dual-energy X-ray absorptiometry (DXA) scanner (GE Healthcare Systems, LUNAR, Madison WI) with the “small animal” software application (software version 8.10). Mice were anaesthetized and placed side-by-side on the scan table and scanned en bloc five consecutive times. Regions of interest (ROIs) for analysis of whole-body composition were manually adjusted once around each mouse and then copied to the four subsequent scans. Mean LBM and FM for each mouse in a group was then calculated from the 5 consecutive scans.

### Hang test

The mouse was placed on a suspended wire for a 3 minute-period. When the mouse fell off the wire time was paused, and the mouse put back on the wire. Deliberate and accidental falls were not included. From an initial score of 10, each fall decreased the score by 1, and the total number of falls over the 3 minute-period was noted as test result [12].

### Glucose tolerance test

Mice were fasted for 3 hours and given an i.p. injection of glucose (2 g/kg body weight) in order to test glucose tolerance (n = 8–9). Blood samples were taken from the tail at 0, 15, 30, 60 and 120 min after glucose injection.

### PCR

Total RNA was isolated from frozen muscle tissue samples (n = 8) by tissue homogenization and RNA extraction using the TRIzol method (Invitrogen). Purity and quantity of the isolated RNA were determined by a Nanodrop spectrophotometer (Thermo Scientific). Total RNA (500 ng) was reversely transcribed into complementary DNA (cDNA) using the High Capacity cDNA Reverse Transcription kit with random hexamer primers (Applied Biosystems). PCR amplification was monitored in real time using the WiiA7 real-time PCR machine (Applied Biosystems) and SYBR green as a fluorescence marker (SYBRGreen PCR Master Mix, Applied Biosystem). Quantification was performed by normalizing to standard curves for each gene. The target genes presented as their ratio to ribosomal 18S RNA.

### Western blotting

Muscle tissue samples were homogenized in a Tris lysis buffer with the use of Qiagen Tissuelyser. Proteins were separated on a 4–15% Criterion TGX Precast Gel (Bio-Rad) by electrophoresis and transferred to a polyvinylidene difluoride membrane (Bio-Rad), using the Trans-Blot Turbo Transfer system at 2.5A–25V for 10 min (Bio-Rad). After blocking with 1% Fish Skin Gelatin (Sigma-Aldrich), the membrane was incubated overnight at 4°C with either anti-Akt (Cell Signalling), anti-phospho-Akt (Ser473, Cell Signalling), anti-mTOR (Cell Signalling) or anti-phospho-mTOR (Ser2448, Cell Signalling) antibodies in 1% Fish Skin Gelatin. Secondary anti-bodies used were HRP-conjugated Polyclonal Goat Anti-Rabbit Immunoglobulins (DAKO) in 1% Fish Skin Gelatin, and proteins were visualised with super signal west femto luminol/enhancer solution (Thermo Scientific). Proteins were analysed by the ChemiDoc XRS system (Bio-Rad) and the Quantity One software (Bio-Rad). Unspecific staining of proteins with Reactive Brown (Sigma-Aldrich) was used as loading and transfer control.

### ELISA

Serum interleukin 6 (IL-6) concentrations were measured by a sandwich ELISA (R&D Systems). The sensitivity was 6.27 pg/ml and the intra-assay coefficient of variation was below 15%. Serum ghrelin concentrations were measured by the Luminex ghrelin singleplex kit (Millipore) in serum samples from the second study. The sensitivity was 5.18 pg/ml and the intra-assay coefficient of variation was below 10%.

### Intramuscular ion and glycogen content

For ion content, total Na^+^, K^+^ and Mg^2+^ were extracted by trichloroacetic acid, and Mg^2+^ content was determined by atomic absorption spectrophotometry (Solaar AAS, Thermo), while Na^+^ and K^+^ were determined with a Radiometer FLM3 flame photometer, as previously described [13]. For glycogen content, quadriceps femoris (QF) muscles (n = 8) were acid hydrolyzed in 1 N HCl for 2 hours at boiling point and subsequent neutralized in 1 N NaOH. The liberated free-glycosyl units were determined spectrophotometrically by using FluoroSkan (Ascent, Labsystems) at 355 nm excitation wavelength and 460 nm emission wavelength using software Ascent version 2.4.

### Statistics

For multiple comparisons, statistical analysis was performed using two-way analysis of variance (2-way ANOVA) followed by post hoc tests with Bonferroni corrections. A 2-way ANOVA with repeated measures was used when the effects of experimental factors was analyzed at different time-points. The statistical significance of the difference between measurements (pre- vs. post-treatment) was obtained from a two-tailed paired T-test. Data analysis was performed using the GraphPad Prism version 5.0 software. Results are expressed as means ± standard error of mean (SEM). The criterion for significance was set at a probability of less than 0.05.

## Results

### Exercise ameliorates effects of cisplatin on body weight, lean mass, food intake and strength

**Figure 1:**
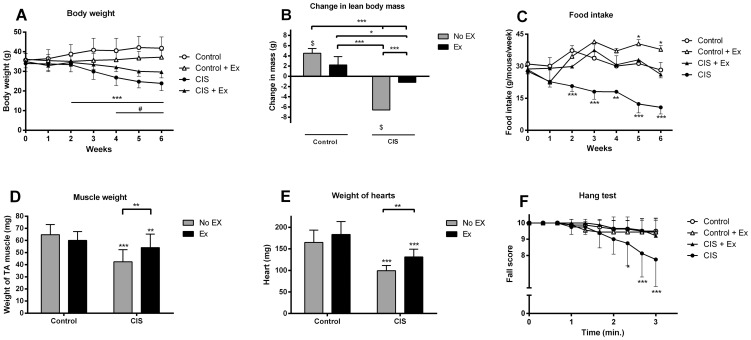
The effect of cisplatin and voluntary wheel running on muscle mass, strength, food intake and body weight. Mice (n = 16–18) were treated with 4 mg/kg cisplatin (CIS) or saline (Control) once weekly for 6 weeks. During treatment mice were assigned to running wheels in their home cages (Ex) or not. A) Body weight was recorded weekly during the intervention. *** Indicates a significant effect of time (change from baseline, P<0.001), # indicates significant different from the control group (P<0.001)(No Ex = 16, Ex = 18). B) Change in lean body mass (LBM) by DXA scanning before and after the 6 weeks intervention (No Ex = 8, Ex = 9). C) Food intake was recorded per cage with 3 mice. A significant effect of time (P<0.001), cisplatin treatment (P<0.001) and the interaction (P<0.001) between the two was observed (n = 6). D) Weight of TA muscle (No Ex = 16, Ex = 18). E) Weight of heart (No Ex = 16, Ex = 18). F) Fall score from hang test performed 3 days after last cisplatin treatment (No Ex = 8, Ex = 9). Results are presented as means ± SEM. Statistical analysis was performed by 2-way ANOVA with repeated measurements and post hoc test (A, C, D) or post hoc paired T-tests (B) with Bonferroni corrections. * P<0.05, ** P<0.01, ***P<0.001 indicates statistical significance compared to non-exercising control or as indicated by the lines except in A) or ^$^P<0.05 compared to pre-treatment levels (B).

Six weeks of cisplatin treatment induced a 29.8% loss of body weight (P<0.001, n = 16) compared to pre-treatment levels in sedentary mice (FIG. 1A), and these mice weighed 43% less than control mice. Evaluations of DXA scans showed that the sedentary cisplatin-treated mice lost 6.6+/−3.6 g of LBM from pre-treatment levels (P<0.001, n = 8 mice, FIG. 1B), which was on average 8.4 g lower in comparison to saline treated mice after treatment. Sedentary cisplatin-treated mice became anorexic, eating less than 50% than saline control mice (P<0.001, n = 6 cages per group, FIG. 1C). Weight reductions of 22.3±2.4 mg tibialis anterior (TA) muscle and 65.4±7.0 mg heart was found in sedentary cisplatin-treated mice compared with controls (FIG. 1D–E), and sedentary cisplatin-treated mice had an average of two falls during the 3 min hang test, compared to no falls in saline control mice (P<0.001, CON: n = 8, EX: n = 9) (FIG. 1F).

Voluntary exercise, by access to running wheels, attenuated weight loss to 15% compared to pre-treatment level in cisplatin-treated mice (P<0.001, n = 18, FIG. 1A), halving the weight loss compared to non-exercising cisplatin-treated mice (P<0.001), but still causing a significant drop in body weight compared to saline-treated exercising mice (P<0.001). Wheel running prevented loss of LBM by maintaining muscle mass in cisplatin-treated mice, while exercising control mice gained 2.2+/−0.5 g LBM during the treatment period (P<0.05, n = 9)(FIG. 1B). Food intake was normalized, as exercising cisplatin-treated mice ate the same amount of fodder as saline treated mice and 58.8% more than the sedentary cisplatin-treated mice (P<0.001, n = 6 cages per group) (FIG. 1C). The weight reductions of TA muscle and heart was attenuated in the exercising cisplatin-treated mice (FIG. 1D–E). Muscle strength was also maintained, as defined by no falls in the hang test (FIG. 1F).

### Cisplatin-induced signalling events in muscle

**Figure 2:**
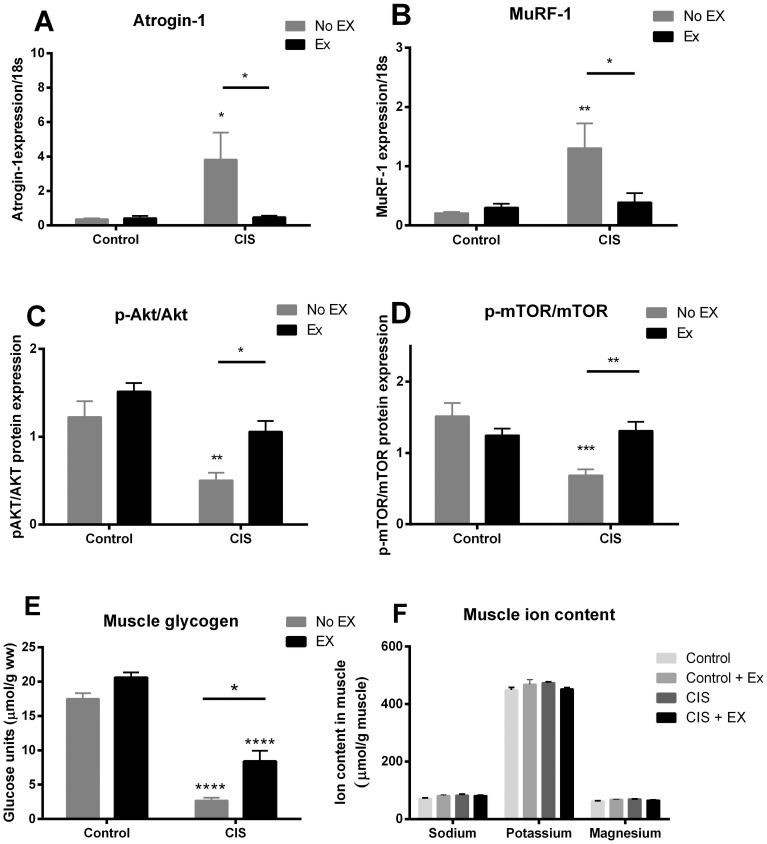
Changes in muscular signalling after cisplatin treatment. Gene expression of A) Atrogin-1 and B) MuRF-1 was determined in muscles from cisplatin-treated (CIS) or saline-treated mice (Control), randomized to exercise training during treatment (Ex). Ratio between phosphorylated and total C) Akt (Ser473) and D) mTOR (Ser2448) was measured by Western blotting. Total-Akt and total-mTOR protein abundance did not differ significantly between groups (data not shown). For pAkt/Akt, a significant effect of exercise (P<0.001) and cisplatin treatment (P<0.0001) was observed in the 2-way ANOVA, while for p-mTOR/mTOR, a significant effect of cisplatin treatment (P<0.001) was observed, (No Ex = 8, Ex = 9). E) Muscle glycogen content, and F) muscular ion content. Results are presented as means ± SEM (n = 8). Statistical analysis was performed by 2-way ANOVA with Bonferroni's post hoc test. *P<0.05, **P<0.01, ****P<0.0001 indicates statistical significance compared to non-exercising control or as indicated by the lines.

In line with the cisplatin-induced loss of muscle mass, we found a 6 and 10-fold up regulation of the two muscle atrophy-related genes, *MuRF-1 and atrogin-1* in the sedentary cisplatin group (P<0.01, FIG. 2A–B). Voluntary wheel running during cisplatin treatment abolished this cisplatin-induced expression of *MuRF-1* and *atrogin-1* mRNA (P<0.05, FIG. 2A–B). By western blotting, we showed reduced phosphorylation of the hypertrophy-related proteins Akt and mTOR in muscles of cisplatin-treated mice (P<0.01) (FIG. 2C–D, FIG. S2). This repression of Akt and mTOR was reversed in the exercising cisplatin-treated mice.

### Muscular energy and ion status

Glycogen content in the cisplatin-treated muscles were 14.8% of the glycogen content in the control muscles (P<0.001). Access to running wheels increased glycogen content in cisplatin-treated mice to 47.8% of the control muscles (P<0.001 compared to control muscles; P<0.01 compared to CIS muscle, FIG. 2E). Cisplatin did not alter muscular water and ion content (FIG. 2F).

### Whole body metabolism and control of inflammation during acute exercise

**Figure 3:**
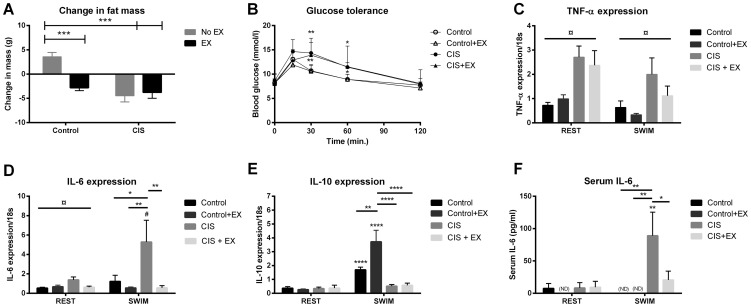
Exercise does not reverse cisplatin induced inflammation and glucose intolerance. After 6 weeks of cisplatin treatment, A) change in fat mass (FM) by DXA scanning, and B) blood glucose levels during a glucose tolerance test, were determined. Before euthanasia, half of the mice performed a single bout of acute exercise (60 min swimming). Gene expression of C) TNF-α, D) IL-6 and E) IL-10 and F) serum IL-6 in mice euthanized before swimming (REST) or immediately after the swimming exercise (SWIM). ND: Values below detection rate. Results are presented as means ± SEM (n = 8). Statistical analysis was performed by 2-way ANOVA with Bonferroni's post hoc test and post hoc T-test for the CIS group. ¤ indicates cisplatin treatment effect (P<0.05) by 2-way ANOVA. *P<0.05, **P<0.01, ***P<0.001, ****P<0.0001 indicates significance in post hoc tests indicates compared to non-exercising control or as indicated by the lines.

Both cisplatin-treated groups lost fat mass (FM) (P<0.01, CON: n = 8, EX: n = 9) (FIG. 3A), and showed significant impairment in glucose tolerance (FIG. 3B). These effects were unaffected by voluntary wheel running during cisplatin treatment. Glucose tolerance can be impaired by local muscular inflammation. In line with this, we found that cisplatin treatment induced TNF-α and IL-6 mRNA expression (P<0.01, n = 8) (FIG. 3C+D). Exercise during cisplatin attenuated the cisplatin-dependent induction of IL-6, but this could not prevent induction of TNF-α.

Acute exercise is known to have direct anti-inflammatory effects. To test this response after cisplatin treatment, half of the mice performed a 60 min swimming bout. The acute exercise induction of IL-10 mRNA expression observed in control mice was completely blunted in cisplatin-treated mice (P<0.05, n = 8). Voluntary wheel running during the cisplatin treatment did not rescue the acute exercise induction of IL-10 (FIG. 3E, n = 9), potentially explaining why exercise did not prevent muscular inflammation. We found a 4-fold induction of IL-6 mRNA expression and 12-fold increase in serum IL-6 in cisplatin-treated mice following swimming (P<0.01 and P<0.001 respectively, n = 8, FIG. 3D+F), while wheel running tended to attenuate this induction (P = 0.102, n = 9, FIG. 3D+F). The induction of IL-6 is highly dependent on glycogen depots in the muscles, and correlates with the reductions in these during the 1 hour of swimming.

### No effect of antiemetic drugs on food intake

**Figure 4:**
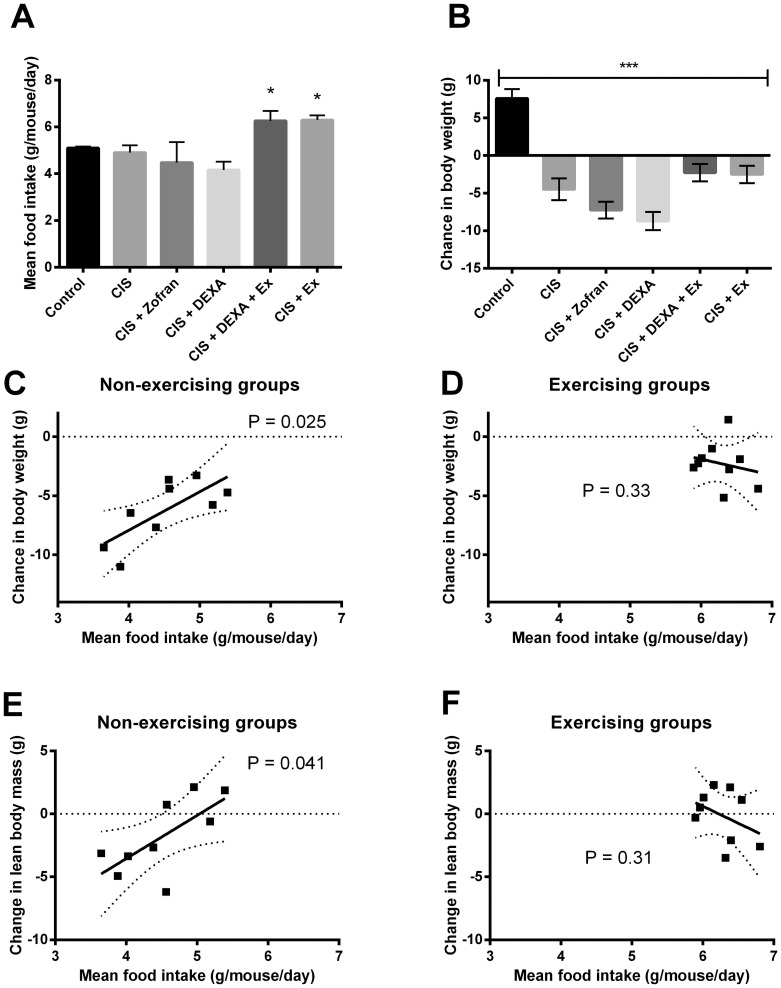
Effect of anti-emetic medication on body weight and mean food intake. Mice were treated with 4 mg/kg cisplatin (CIS) or saline (Control) once weekly for 6 weeks and received concomitant ondansetron (Zofran) or dexamethasone (DEXA) for 3 consecutive days after each cisplatin treatment. A) Mean food intake, and B) change in body weight. Statistical analysis was performed by oneway ANOVA with Bonferroni's post hoc test (Mean ± SEM, N = 10). *P<0.05, ***P<0.001 compared with Control mice. Linear regression analyses between mean food intake (calculated from 3–5 cages per group) and change in body weight (C+D) (mean of mice in each cage), or change in lean body mass (E+F) (mean of mice in each cage) in non-exercising cisplatin-treated groups (CIS, CIS+Zofran and CIS+DEXA) and exercising cisplatin-treated groups (CIS+DEXA+EX and CIS+EX) were performed.

**Figure 5:**
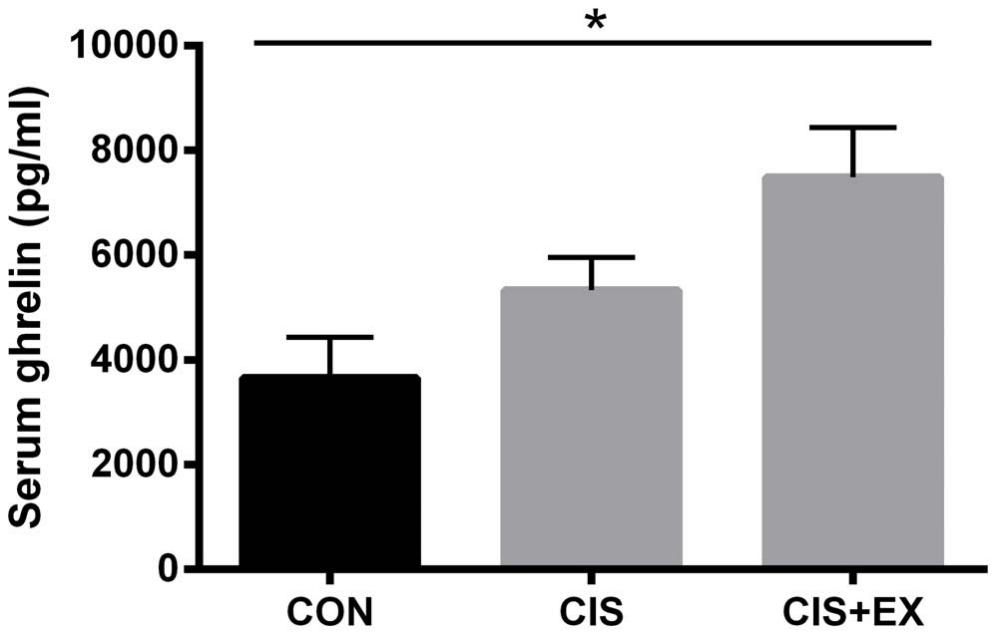
Exercise during cisplatin treatment stimulates the appetite hormone ghrelin. In the control, non-exercising cisplatin-treated (CIS) and exercising cisplatin-treated (CIS+EX) groups from fig. 2, serum ghrelin was determined by ELISA. Statistical analysis was performed by 1-way ANOVA with Bonferroni's post hoc test. *P<0.05 indicates significance in post hoc tests as indicated by the line.

As exercise was shown to normalise food intake in the cisplatin-treated groups, we investigated if anti-emetic drugs would influence food intake. I.p. administration of the antiemetic drugs (ondansetron or dexamethasone) was not able to normalize food intake or prevent loss of body weight in this model (FIG. 4A–B). In addition, we found a significant correlation between decreased food intake and loss of body weight (R^2^ = 0.53, P = 0.025, FIG. 4C), as well as loss of LBM (R^2^ = 0.47, P = 0.041, FIG. 4E) in the non-exercising groups. These correlations dissipated in the exercising groups (FIG. 4D+F). Stressing the effect of exercise on food intake, we found that the appetite hormone ghrelin was up-regulated in the exercising cisplatin-treated groups (7491+/−948 pg/ml, n = 18) compared to non-exercising cisplatin-treated mice (5333+/−622 pg/ml, n = 24) and saline-injected controls (3664+/−777, n = 9, P<0.05) (FIG. 5).

### No observable effect of exercise on muscular mass restoration after cisplatin treatment

**Figure 6:**
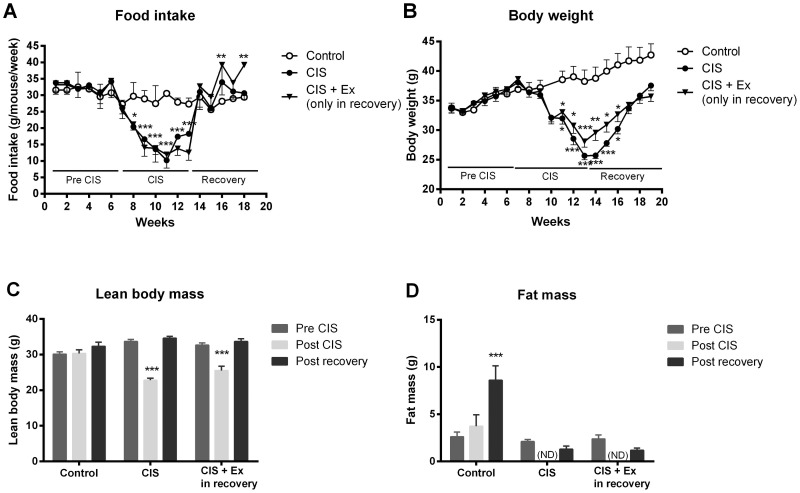
Muscle restoration in cisplatin treated mice. To evaluate the regenerative response, mice were treated weekly for 6 weeks with cisplatin (4 mg/kg), and then followed for additional 6 weeks. A) Body weight and B) food intake was recorded weekly. C) Lean body mass (LBM) and D) fat mass (FM) was determined by DXA scanning before cisplatin treatment (pre CIS), immediately after the 6 weeks cisplatin treatment (post CIS) and after 6 weeks recovery (post recovery). ND: values below detection limit. Results are presented as means ± SEM. *P<0.05, **P<0.01, ***P<0.001 indicates significant difference compared to baseline control.

In order to evaluate muscle mass restoration, mice were followed for a 6 week recovery period after the initial 6 weeks of cisplatin treatment. During the recovery period, mice returned to pre-treatment levels of body weight and food intake (FIG. 6A–B), and regained all lost LBM and fat mass as determined by DXA scanning (FIG. 6C+D). Exercise during the recovery phase had no additional effect on muscle mass restoration after cisplatin treatment (FIG. 6A–C). In addition, cisplatin no effect on satellite cell proliferation (data not shown), nor was myoD gene expression affected by cisplatin treatment or exercise (data not shown).

## Discussion

The present study explores the capacity of exercise to counteract cisplatin-induced adverse effects in skeletal muscle. There were two principal findings: 1) cisplatin treatment induced muscular dysfunction as defined by reductions in muscle mass and strength, and impairments in insulin resistance and inflammation. 2) Voluntary wheel running could effectively abrogate cisplatin-induced loss of muscle mass and strength, as well as attenuate intramuscular signalling events, in parallel with normalization of body weight and food intake. Yet, voluntary wheel running had no protective effect on impaired glucose tolerance or inflammatory status. In concert, these findings provide novel evidence for both direct and indirect effects of cisplatin on muscular function, as well as elucidating the capacity and limitations of exercise as therapeutic countermeasure for cisplatin-induced muscle dysfunction.

### Protective mechanisms of voluntary exercise in mice

In our cisplatin-treated mice, we found severe reductions in lean mass, which mostly likely was driven by reduced food intake, given the close correlation between reduction in food intake and the level muscle wasting during the treatment period. Importantly, voluntary wheel running normalized food intake, preserving muscle mass and function. Exercise is generally recognized for increasing appetite, explaining why exercise interventions in overweight individuals do not always result in the expected weight loss. Conversely, limited information is available on regulation of appetite by exercise in anorexic or undernourished states. In our mouse model, we found that exercise increased the systemic levels of the appetite hormone, ghrelin, indicating that exercise induces direct hormonal effects on food intake in these anorexic mice. In an attempt to control the energy intake, we investigated the concurrent effect of anti-emetic drugs. Neither i.p. dexamethasone nor ondansetron, two agents commonly administered as supportive care medication, improved food intake in our mouse model. Thus we cannot in the present study separate the effects of exercise from the derived effect of increased food intake.

### Irreversible muscular inflammation and insulin resistance

Cisplatin treatment has been associated with substantial systemic and nephritic inflammation [14]. After 6 weeks of cisplatin treatment, we observed a marked induction of TNF-α expression in the muscles, which was not reversed by exercise. This discrepancy between TNF-α expression and loss of LBM indicates that inflammation-driven muscle wasting is not an important pathway in cisplatin-induced muscle wasting in our model. TNF-α can impair glucose uptake by altering insulin signal transduction in skeletal muscle, as shown through infusions of TNF-α at physiological relevant systemic concentrations in healthy humans [15]. These findings may explain the impaired glucose tolerance in both the sedentary and exercising cisplatin-treated groups.

Training comprises of repeated bouts of acute exercise, during which the systemic concentration of anti-inflammatory cytokines i.e. IL-1ra and IL-10 increases, driving the anti-inflammatory response of exercise [16]. During an acute bout of exercise, we observed that the induction of IL-10 mRNA was completely blunted in both cisplatin-treated groups. This lack of release of anti-inflammatory cytokines during acute exercise, may explain why TNF-α could not be controlled by voluntary wheel running in the exercising cisplatin-treated group.

In contrast, IL-6 expression increased during an acute bout of swimming in both the sedentary groups, yet most pronounced in the cisplatin-treated group. This response was completely blunted in both exercising groups. IL-6 expression in the muscle is highly dependent on the glycogen content of the muscle, and the induction of IL-6 during acute exercise is a sensor of carbohydrate availability. Thus exercise-induced release of IL-6 can signal from the contracting muscle to other organs, increasing hepatic glucose output and gluconeogenesis, as well as lipolysis in the adipose tissue [17]. In line with this, we found that induction of IL-6 expression correlated with glycogen depletion in the muscles of cisplatin treated mice.

### Exercise as muscle preserving intervention in cancer patients

Exercise intervention is increasingly being promoted in cancer patients, as numerous trials have proven safe, feasible and efficient in improving functional capacity, fitness, lean mass and strength, as well as psychosocial parameters [7], [18], [19]. Most studies have been performed in patients with breast and prostate cancer, yet studies in patient groups receiving cisplatin-based therapy are emerging [20], [21], [21]–[23].

Traditionally exercise is divided into endurance training with focus on improving cardiovascular output, fitness levels and energy expenditure, and strength training concentrating on building muscle mass [10]. Both modalities have been tested in cancer patients undergoing chemotherapy, showing that they can improve functional capacity and muscle mass [7]. For patients receiving cisplatin treatment, the urgent concern for restoring muscle mass implies that strength training would be an effective intervention in this group. Yet for the sedentary cancer patient any engagement in physical activity would stimulate muscular hypertrophy signals and based on the results here stimulate food consumption.

In this study, we chose to use the voluntary wheel running model of exercise. This model, in contrast to other rodent exercise regimens, is entirely unforced as the mice run at night at their own pace and will. This model has the benefit of inducing minimal stress, or no stress at all, with high exercise performance.

This study supports a general recommendation for exercise in patients receiving cisplatin-based treatments. Our results show that even under anorexia and energy-depleted states, exercise not only prevents muscle wasting, but normalizes energy intake possibly through a direct effect on the appetite hormone ghrelin. In addition, both the numerous exercise trials in cancer patients and controlled exercise trials in mice clearly demonstrate that exercise does not have any tumour promoting effects, if anything exercise seems to decrease tumour growth through yet unidentified mechanisms [24]–[26]. These findings can have relevance for daily clinical practice since weekly administration of cisplatin is used routinely, e.g. during radiotherapy of head- and neck, and cervical cancer [27], [28]. There has been considerable focus on treatment of nausea and emesis. Yet maintenance of appetite is an equally important factor, and this study points out that exercise may independently improve appetite.

### Conclusion

In summary, we found that exercise during treatment could alleviate cisplatin-induced muscle wasting and signalling impairments in protein degradation and hypertrophic pathways in healthy mice. Thus exercise training might be a promising strategy for preservation of muscle mass in cancer patients receiving treatment with cisplatin, and holds the potential for improving physical capacity, quality of life and overall survival.

## Acknowledgments

Anne Boye, Lone Christensen, Henriette Pilegaard and Vibeke Uhre are acknowledged for their technical assistance. Our dear colleague Hanne Gissel unfortunately passed away in the final phases of the preparation of this article.

## Supporting Information

Figure S1 **Running distance was evaluated weekly in the groups of experiment 3 (N = 3 cages).** Statistical analysis was performed by 2-way ANOVA with Bonferroni's post hoc test. *P<0.05, ***P<0.001 indicates significance in post hoc tests from the control group.
(TIF)

Figure S2 **Representative western blotting bands of the data presented in** **FIG. 2C-D** **of phospho-Akt (p-Akt), total Akt, phospho-mTOR (p-mTOR), and total mTOR, as well as the whole gel stained with reactive brown as loading control.**
(PDF)

Checklist S1 **ARRIVE guideline checklist.**
(DOC)
