# Supplementary figures and images for: Voluntary Exercise Prevents Cisplatin-Induced Muscle Wasting during Chemotherapy in Mice
Source: PLoS One. 2014 Sep 30;9(9):e109030. doi: 10.1371/journal.pone.0109030 (PMC4182656; doi:10.1371/journal.pone.0109030)

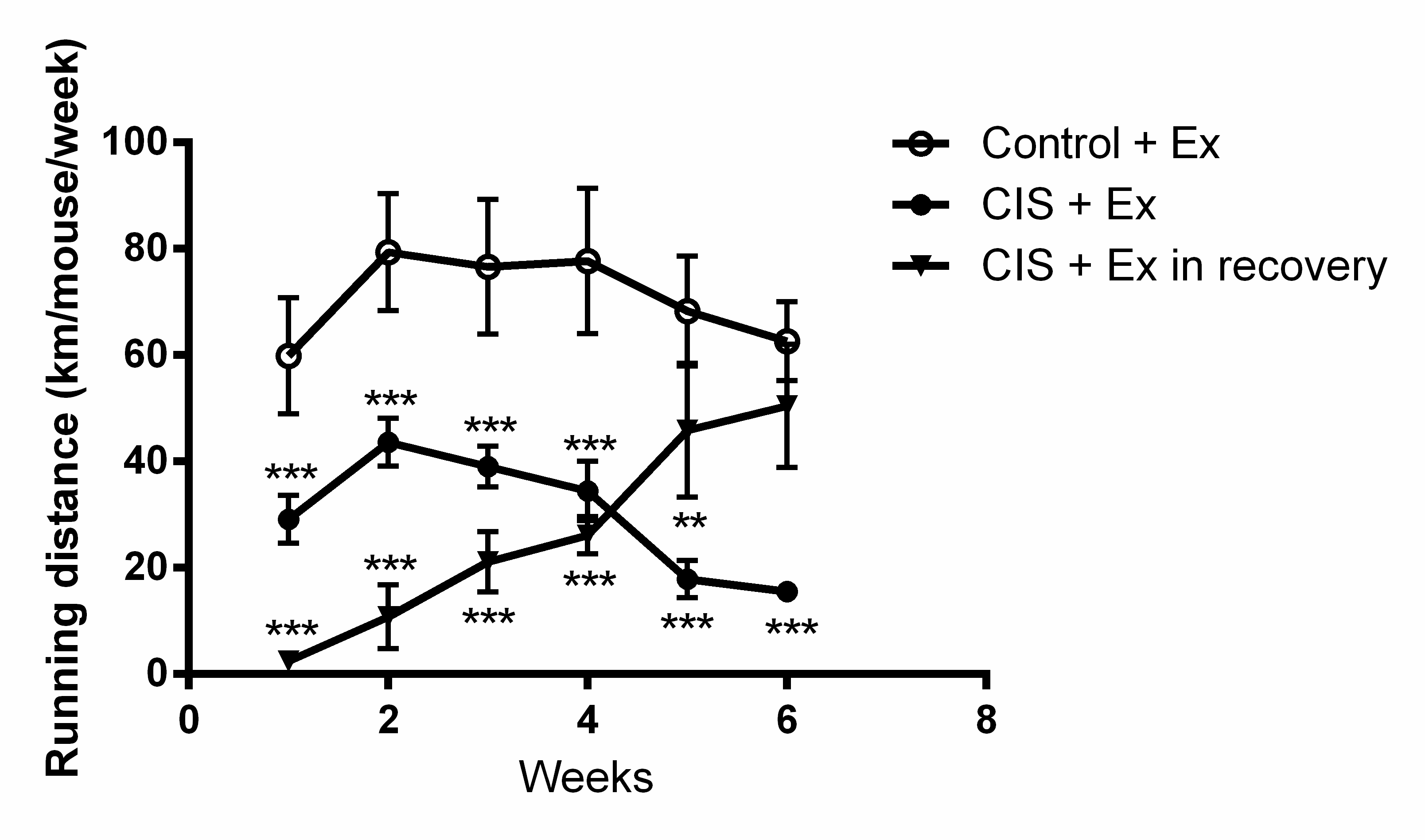

Supplement: Figure S1 — Running distance was evaluated weekly in the groups of experiment 3 (N = 3 cages). Statistical analysis was performed by 2-way ANOVA with Bonferroni's post hoc test. *P<0.05, ***P<0.001 indicates significance in post hoc tests from the control group. (TIF) [file pone.0109030.s001.tif]

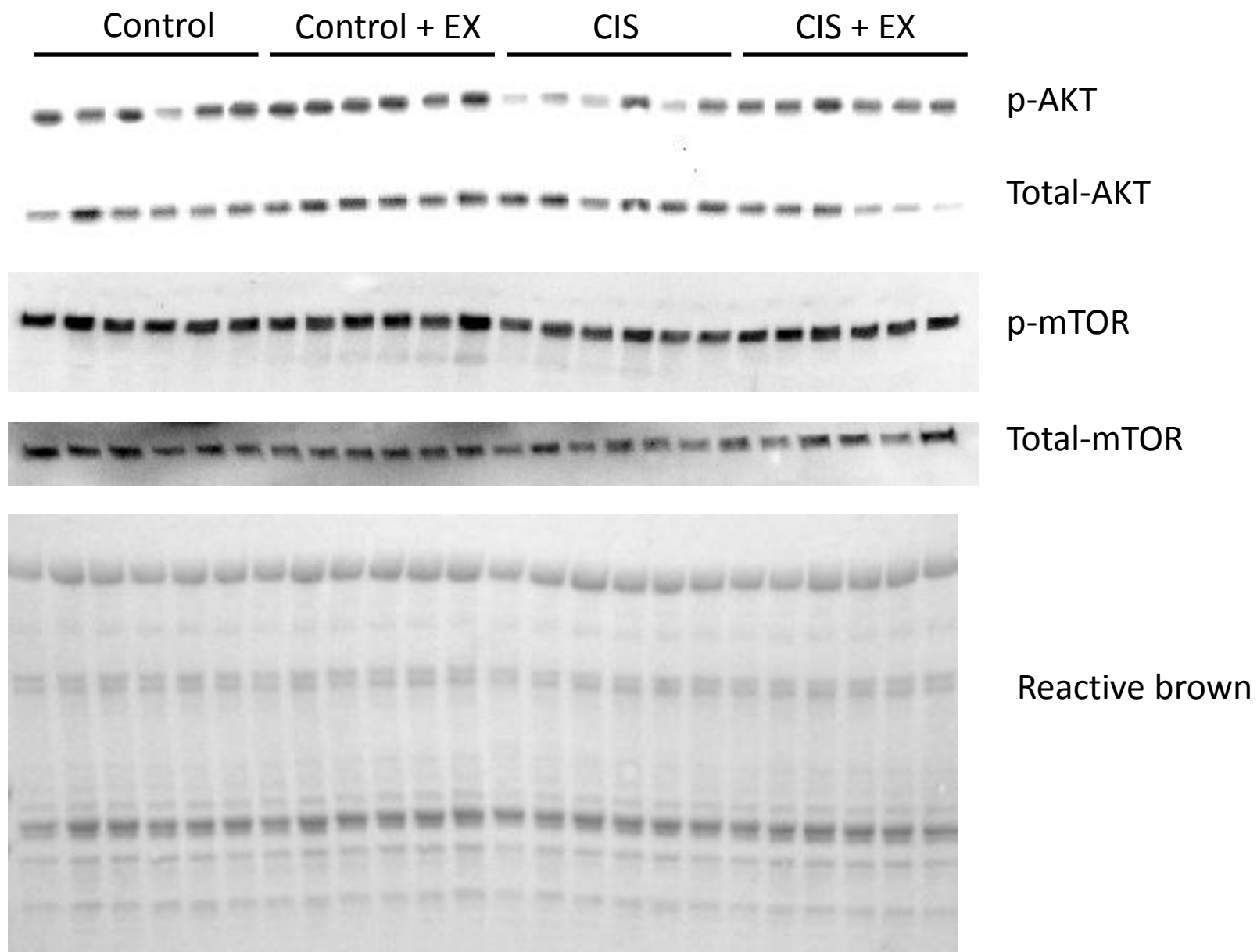

Supplement: Figure S2 — Representative western blotting bands of the data presented in FIG. 2C-D of phospho-Akt (p-Akt), total Akt, phospho-mTOR (p-mTOR), and total mTOR, as well as the whole gel stained with reactive brown as loading control. (PDF) [file pone.0109030.s002.pdf]
